# Audio-diary reflections after community focus groups to address local racial inequities in the neonatal intensive care unit

**DOI:** 10.1038/s41372-024-02176-y

**Published:** 2024-11-15

**Authors:** Kayla L. Karvonen, Olga Smith, Brittany Chambers-Butcher, Patience Afulani, Tameyah Mathis-Perry, Khuzaima Rangwalla, Monica McLemore, Elizabeth E. Rogers

**Affiliations:** 1 Department of Pediatrics, University of California San Francisco, San Francisco, CA USA; 2 California Preterm Birth Initiative, San Francisco, CA USA; 3 Independent Researcher, Antioch, CA USA; 4 Department of Human Ecology, College of Agricultural and Environmental Sciences, University of California Davis, Davis, CA USA; 5 Department of Epidemiology and Biostatistics, University of California San Francisco, San Francisco, CA USA; 6 Morehouse School of Medicine, Atlanta, GA USA; 7 University of California San Francisco School of Medicine, San Francisco, CA USA; 8 Department of Child, Family, and Population Health Nursing, University of Washington, Seattle, WA USA

**Keywords:** Paediatrics, Health services

## Background

Extensive literature highlights the persistent racial inequities experienced by families in the neonatal intensive care unit (NICU) [1]. Scholars have recommended community-based approaches to addressing racial inequities, such as focus groups that invite community members to exchange ideas and express opinions [2, 3]. Focus groups are designed to disrupt power hierarchies between researchers, moderators, and participants, encouraging participants to share stories, articulate perspectives on social and health issues that affect them, and recommend strategies to address them that are grounded in lived experience, transforming participants into agents of change [3].

In the Racial and Ethnic Justice in Outcomes in Neonatal Intensive Care (REJOICE) study, we previously highlighted serial focus groups with staff and parents to address racial inequities in the NICU [4]. In this study, we aimed to understand the impact of the focus groups using follow-up audio diaries.

## Methods

As detailed in the previous article [4], semi-structured focus groups were conducted in 2022-2023 in an urban level 4 NICU with 8 multidisciplinary staff and 8 Black and Latinx parents with children previously hospitalized in the NICU [4]. Following the focus groups, participants were offered to conduct audio diaries on their phones or audio recording devices. Six out of 16 focus group participants recorded audio diaries. All participants were female, and identified as Black, Latinx, and Asian. Instructions for the audio-diary guide included 10 structured questions (Supplementary Appendix). Audio diaries were recorded, transcribed, and uploaded into Dedoose software. Thematic analysis was utilized to analyze the data. Three researchers developed and applied the codebook to transcripts, generated themes independently, and met together with the larger study group to discuss, develop, and organize the themes.

## Results

Four themes were developed (Table 1).

**Table 1:** Themes and exemplary quotes.

Themes	Exemplar quotes
Bidirectional shared empathy	“I hear that everybody in this room genuinely has the same heart but has a totally different understanding from person to person of experience. Her fear was retaliation. I was retaliated on. And it’s just like the providers that had to sit and watch. …So that’s important to me also and that makes me sad for providers and I, as a parent, I still want to know how can we support the providers also because they’re here. They have to still be here.”
	“I think the most meaningful thing that was discussed was how the providers felt about the ICN and how they feel that they’re being treated. I think it’s a good thing to hear because we don’t know what their lives are and how they get treated and it just puts everything into perspective that they are also human too, you know? They are sitting here taking care of these fragile little beings. They have emotions too and it was really good to hear that.”
	“Everything, everything, everything, truthfully, has been perfect for me. Eh… I liked it a lot, I am happy to attend the group. I hope that it continues, I hope that they keep taking us into account. Eh, we liked it a lot. We liked it a lot, eh… giving our opinion, and… and, well, talking about… about… about all our experiences and all the situation[s] we live in. It is something that is very – it is something very gratifying.
Power sharing	“The power was definitely shared by the parents. There was this one parent, man, she was going in. She was just like not holding back and she was just saying her truth and it was like, “Wow.” I’m over here trying to navigate how to say things, you know what I mean, like what to say, how to make it make sense. It makes sense to me, so other people could understand and trying not to say, oh, I don’t want to say this. You know, I don’t know. But when one of the parents was just like saying it raw and uncut like, you know, unfiltered. I was like, “Man, that is powerful. That is powerful. I like that.”
	“Man. It was definitely a powerful session and definitely started something great. I feel like this will go somewhere. This is not going to stay small. This is going to blow up. And this is great and I’m happy to be a part of it really. I am…I thought this was a great session. Just the beginning but man, I felt a lot of fire. Things were going. People were speaking. Things were being said, you know? We were opening up to conversation. It was awesome, really.”
Validation	“I thought it went well. It was good to know that there were a lot of other families that went through the same things that I went through and that I wasn’t crazy. It was just nice to hear.”
	“I was able to just hear a lot of different people’s stories and just compare them to mine based on our experiences and where we are or how we were dealt with in the setting or just being with UCSF. Me meeting with other parents made me realize that I’m not alone in how I feel my feelings are valid.”
Therapeutic effect	“I felt it very therapeutic and kind of relieving. It felt good to listen to other moms talk about their journeys and what they experienced in the ICN. It took me a while to reflect on them [my emotions]. I had a day to myself the day after to kind of work on them just because it brought up a lot of old trauma I guess from the ICN that I hadn’t worked on yet but it helped. It helped a lot.”
	“My raw feeling did hit me in the gut during the session which left me kind of speechless throughout the whole session. But also my raw emotions made me feel like I needed to do something or do better. I am more aware of my surroundings at beside toward the parents versus when before I was so task-oriented and would walk into the room and to the bedside and attend to the patient without making sure I explained to the parents first or even taking the time to listen and acknowledge them.”

### Theme 1: Bidirectional shared empathy

Whereas the focus group discussion was centered initially on parent experiences with racism and discrimination in the NICU, parents also learned about staff experiences. Although staff and parent roles are distinct, both groups shared experiences of trauma, emotional tolls, and fatigue while in the NICU. Parents and staff found meaning, new realizations, and appreciation for each other and their roles when they reflected on humanism and intentions. Parents noted differences in staff perspectives, yet shared goals of desiring the best outcome for the child. This newfound shared empathy deconstructed parents preconceived notions of the apathy of healthcare staff and promoted shared bidirectional recognition of humanity.

### Theme 2: Power sharing

While reflecting on the focus group sessions, participants described the feeling of power sharing and deconstructed hierarchies of power. Power is the possession of control or authority, and in this case, applies to participants speaking up and advocating for change [5]. Parents were encouraged to speak up after witnessing other parents share their raw and uncut experiences and truths. These observations fueled optimism and hope among participants. Participants described the focus groups as catalysts for change.

### Theme 3: Validation

Parents described the NICU as an isolating place in both their racialized experiences and traumatic hospitalization experiences. Focus groups were perceived as a place for validation of comparing their similar but unique experiences. Validation, meaning acknowledging, and accepting another person’s experience or world-view, was positive, healing, and combated prior sentiments of isolation, doubt, and feeling “crazy” as a result.

### Theme 4: Therapeutic effect

Through bidirectional shared empathy, power sharing, and validation, participants described therapeutic, relieving, authentic, and powerful experiences. The focus groups evoked a strong emotional response and catalyzed a commitment for actionable change. Staff described concrete next steps for changing their behaviors. Reflection continued beyond the sessions and provided a unique opportunity to revisit unaddressed emotions and to advocate for families in the NICU.

## Discussion

In this study, focus groups with NICU parents and staff not only provided insight into addressing racial inequities [4] but were also perceived as therapeutic through bidirectional shared empathy, power sharing, and validation.

The focus group study uniquely brought a diverse group of NICU staff and parents together to share their perspectives on racial inequities [4]. The study design promoted hierarchy disruption: first, by bringing individuals in proximity across both racial and traditional medical staff/parent hierarchies; second, by including an initial power-sharing session for parents. Although power-sharing was intentionally manifested, the bidirectional shared empathy, validation, and therapeutic impact experienced by staff and parents were unanticipated. Future studies should explore focus groups functioning as peer support groups, a community-based recommendation to address racial inequities [4]. Parents were invited to participate after NICU discharge, but future studies of interventions during their child’s hospitalization may also be impactful across these four themes. The major limitation of this study is the small sample size and incomplete participation. Non-participating staff and parents may have had unique perceptions of the peer support groups, not captured here.

## Conclusion

Utilizing audio-diary data, we found that focus and peer support groups are potential mechanisms by which to address racial inequities in the NICU through power sharing, bidirectional shared empathy, validation, and ultimately a therapeutic effect.

## Supplementary information

Supplementary Appendix

## Data Availability

Individual participant data from audio-diary transcripts will not be shared. Even when data is de-identified, there is significant risk of identifying participants and their responses, given the small sample size in a small patient and workplace community.
