# Supplementary material for: Audio-diary reflections after community focus groups to address local racial inequities in the neonatal intensive care unit
Source: J Perinatol. 2024 Nov 15;45(3):392–4. doi: 10.1038/s41372-024-02176-y (PMC11888981; doi:10.1038/s41372-024-02176-y)
Supplement: Supplementary file 1 — Supplementary Appendix [file 41372_2024_2176_MOESM1_ESM.docx]

Supplemental Appendix: REJOICE Study: Audio Journal Semi-Structured Guide

**Purpose:** I have asked you to complete an audio journal entry today because I want to hear about what it is like for you in the ICN community working group. We are hoping these audio journals will teach us how to be better community members to our families who receive care in the ICN.

**Confidentiality:** Before we begin let’s review some general rules of understanding

- There are no right or wrong answers.
- I just want to know what you think and your opinions.
- Please be honest. You won’t hurt my feelings or affect me in any way.
- I will not ‘tell’ on you if you say something negative.
- If you need to take a break, you can pause the recording, or you can stop at any time

This audio journal entry will be recorded and only the researchers will hear this recording. No one from the care team will be able to trace anything said in this conversation back to you. Nothing you say in this journal entry will impact your baby’s care in a negative way. Before we begin, what would you like your pseudonym to be: _________________? The same pseudonym should be used for all journal entries.

**Begin:** Step 1- First, press the button on your recording device to start recording your audio journal entry. Step 2- Then, state your pseudonym (the nickname you give yourself for the purposes of the audio journal entries) into the device. Step 3- Next, read the question into the recorder that you are answering. The questions are listed further down in these instructions. Step 4- answer the question with your own opinion. Step 5- repeat steps 2 through 4 for the remaining questions until you have answered all ten questions. Step 6- once all questions are answered, press the button on your recording device to stop recording.

1. Describe your overall sense of today’s working group session.
2. What kind of emotions did today’s session evoke for you?
3. How did you work through those emotions?
4. Share at least one thing that was meaningful for you in today’s session.
5. Share at least one thing that was difficult for you in today’s session.
6. In what ways do you feel power was shared by all team members in today’s session?
7. Consider how the researchers facilitated and moderated the meeting, how could they have improved? (prompts: too much academic language, content was confusing, expectations of you were unclear)
8. Describe your understanding of the content shared in the working group session.
9. Share at least one thing you think could improve the sense of community in the working group session.
10. Finally, please share any additional thoughts about today’s session you may have.

**Finish:** Step 1- confirm your recording has been saved either in your phone, on the audio recording device provided by the REJOICE study team or on your computer. Step 2- send a copy of the recording to the REJOICE study team either by email at rejoice@ucsf.edu or in person by returning the actual audio recording device with the recording on it to Olga Smith, Co-Principal Investigator on the REJOICE study. Step 3- receive your pre-activated Visa gift card in the amount of $30.00. Step 4- make sure you have a recording device for your next audio journal entry.
